# “A shoulder to lean on during your first year”—An exploration into a Canadian post-secondary institution’s peer mentor program for varsity student athletes

**DOI:** 10.1371/journal.pone.0298806

**Published:** 2024-05-08

**Authors:** Kathryn Johnston, Far Mutaj, Mandy Frake-Mistak

**Affiliations:** 1 School of Kinesiology & Health Science, York University, Toronto, Ontario, Canada; 2 Faculty of Liberal Arts & Professional Studies, York University, Toronto, Ontario, Canada; University College Dublin - National University of Ireland: University College Dublin, IRELAND

## Abstract

The transition period from high school to post-secondary can be particularly challenging for many, including varsity student-athletes (SAs). To better support SAs through this transitional experience, some institutions have created peer mentor programs. What is unclear, however, is the perceived value of these mentorship programs from the perspectives of multiple stakeholder positions. This paper contributes to the Scholarship of Teaching and Learning by presenting findings of a program evaluation that investigated the perceived value of a peer mentor program to its stakeholders. To accomplish this, semi-structured interviews were conducted with 30 participants to discuss SA’s experiences with being a first year student, making the transition from high school to post-secondary studies, and also, to discuss their lived experiences with the peer mentor program developed for SAs. Using the findings from the inductive thematic analyses, the peer mentor program’s effectiveness, areas of strengths, and areas of improvement are discussed to better align with the stakeholders’ needs and experiences. Findings offer insights into a) the trials and tribulations of the first year SA experience, b) how peer mentor programs can better support SA’s transition to post-secondary education, c) the benefits of conducting a program evaluation, and d) strategies to enhance the peer mentor program to better support students’ needs.

## Introduction

The transition from high school to post-secondary education can be an exciting experience, offering students a chance to build and strengthen friendships, to explore academic disciplines, and to take part in extra- and co-curricular activities [1, 2]. That said, this transition period may also be a time where students feel overwhelmed by newness, experience extreme pressures to perform academically, feel challenged by financial responsibility, and experience feelings of social disconnection and isolation [3, 4]. These examples (amongst many others that have been [and yet to be] studied) shed light on both the complexity and the degree of nuance that likely exists for students during these transition periods, and indicates a clear area of need for research explorations to better understand ways to support students’ needs [5–12].

Importantly, previous work in the field has investigated the transition experiences of different student communities including indigenous students [13, 14], BIPOC students [15, 16], members of the 2SLGBTQUIA+ community [17, 18], first generation students [1, 19–21], international students [22, 23], and student-athletes (SAs) [10, 24–29]. Each of these communities likely has unique and distinct pressures during the transition from high school to post-secondary education, especially those who are at the intersection(s) of these communities [30]. Vaquero-Cristóbal and colleagues [30], who identified SAs with disabilities, were more likely to perceive barriers (and with a greater extent) towards achieving an effective balance between studying and training than SAs without a disability. While it is important that each of these communities listed above (amongst many others) are at the forefront of discussions when understanding student experiences and student support, for the present investigation, we focus on students who are also varsity athletes (SAs).

SAs represent their post-secondary institution in organized, competitive sport, and complete their academic and athletic requirements on a full-time basis. It could be argued that this student sub-group experiences unique performance pressures–spanning both academic and athletic spheres—at the post-secondary level, as their athletic performance is tied to their academic performance (both from an eligibility perspective, and often, from a financial perspective with incentives like scholarships and awards) [24, 31, 32]. As noted in the work by Condello and colleagues [33], which is further echoed in the statement released by the National Collegiate Athletic Association (NCAA) [34], multiple stakeholders providing multi-faceted care through a well-structured and systematic program are essential for supporting SAs with both their athletic and academic pursuits.

In recognizing these significant pressures and challenges, many post-secondary institutions offer structured programming for SAs, often in a peer mentorship design, where experienced or older students are paired with younger, or less experienced students to provide support and guidance in various aspects of their academic and personal lives [35–38]. These peer mentoring programs (PMPs) are varied in terms of their funding, structure, and delivery models, ranging from formal, funded, and curriculum-guided programs, to more informal and volunteer-based mentorship opportunities. While the scale, design, goals, and intentions of these PMPs are institutionally- and departmentally-specific, some of the more common goals of PMPs include, a) enhancing the student experience, b) increasing academic performance, and c) augmenting retention and graduation rates [39]. With these goals in mind, PMPs may not only assist students academically by working to build and strengthen fundamental learning skills required to succeed in a university setting, but they may also allow for the development of meaningful relationships to support students’ social and emotional well-being [40, 41]. For instance, Collier [40] noted potential benefits of incorporating PMPs specifically for SAs, which included a) an increased awareness of institutional resources and support services, b) increased feelings of a sense of belonging, and c) increased perceptions of their ability to excel in academic pursuits. While these benefits have been studied in the context of SAs, PMPs have the potential to support many other student communities [42–44].

Limited research has been conducted on the efficacy of PMPs for SAs in the field [40], and important questions from the lens of Teaching and Learning, remain unanswered. As questions these are likely top of mind for both researchers and practitioners hoping to improve support systems for students, the present investigation seeks to illuminate *the perceived value(s) of a PMP for SAs across various stakeholder positions*. To help address this primary research question, we share our findings from our evaluations of a PMP designed for SAs, to illuminate the perceived impact of the PMP, and to determine whether the goals and intentions of the program met the needs of its stakeholders (i.e., first-year students, peer mentors, staff, coaches, faculty members and Teaching Assistants [TAs] for first-year courses) at the time of the interview, within the context of a large university located in south-western Ontario, Canada.

### The peer mentor program

The PMP under examination was created in 2011 to improve the SA experience during the transition from high school to university. Additionally, in recognizing unsatisfactory first year attrition rates and graduation rates within the SA population, it further acted as the impetus for the program’s development. In an attempt to disrupt those patterns, a PMP was created to a) provide a formal setting for first year SAs to learn and develop academic competencies through an established curriculum, b) create a safe and comfortable space that fosters deep social connections, c) to connect students to on-and off-campus resources, and d) to have fun (Athletic Department at institution, internal presentation, 2016 [45]).

Since 2011, the program has grown and evolved in many ways. This growth is perhaps most evident in the size and scale of the program at the time of the investigation compared to its inaugural year in operation (i.e., 2011). For example, what started in 2011 as a PMP for a few eligible first SAs meeting an academic inclusion criteria (i.e., a grade point average [GPA] threshold) who were paired with a few upper year students informally (i.e., meeting sporadically, either via text, email, or in-person), has since grown to accommodate *all* first year and transfer SAs (i.e., between 100 and 125 students) into a program with nearly 50 mentors and mentors in training (in both paid and in volunteer positions), with multiple full-time and part-time staff members overseeing the delivery of the program. Importantly, for the purposes of the present investigation, the conversations will reflect the PMP at the time of the interviews, which may not necessarily reflect the PMP after that time, as the program is constantly evolving.

At the time of the study, and to accommodate the 100–125 incoming first year SAs, the PMP offered six, one and a half hour sessions each week where students were expected to attend one session each week from September to April (i.e., the fall semester and the winter semester, but not the summer semesters). The first 15 minutes of the session included a check-in between the first year or transfer SA (i.e., the mentee) and the upper year SA mentor (i.e., the mentor). During this time, the check-in focused on three central topics, a) personal (e.g., how the SA was doing and feeling), b) academics (e.g., due dates, grades received, etc.), and c) athletics (e.g., practice and competition updates, potential conflicts, achievements, challenges, etc.). Following the check-in, mentees spent the next 15–30 minutes participating in learning or skill-building workshops covering different topics including a) time management strategies, b) essay writing approaches, c) navigating university resources, d) understanding and supporting wellbeing, and e) effective study techniques. Sessions concluded with a dedicated study and/or work time–a quiet time that allowed both mentees and mentors to work on their studies. It is important to note, that within the institution under investigation, there are many other formal PMPs for a diverse range of student communities beyond the SA group. Many of these programs offer formal peer-to-peer services in terms of one-on-one and group-based check-ins and guided teachings of foundational learning skills.

Upon a first year SA’s acceptance to the university, he/she/they was/were paired with an upper year mentor. To determine the pairing, the first priority was to identify alignment between the mentors’ and mentees’ academic and athletic schedules (taking into consideration their class schedules, tutorials, seminars, labs, and their sport practices, strength and conditioning sessions, mental performance sessions, etc.) during the six available times to choose from. Once a match was identified by schedule availability, the second priority was to match mentors and mentees by academic focus (i.e., matching students by faculty, and ideally by program of study). This was often feasible for the larger academic programs, but became challenging with the smaller and more focused academic programs. In some cases, mentee and mentor pairings would be modified mid-way through the academic year, as some students change their program major, and also because the semester change brought new course and sport schedules that created timing conflicts.

## Methodological considerations

### Transparency and openness

To ensure this research project met transparency and open reporting standards [46], we have cited and provided references for all methods used. All study procedures were approved by the Human Participants Review Sub-Committee, of our institution’s Ethics Review Board (University Research Ethics Board certificate number for approval: STU 2019–442). Written informed consent was obtained from all participants (between March 1, 2020-Jan 1, 2021) prior to their respective interviews. The data and research materials that support our findings are not publicly available to ensure participant privacy. That said, they can be made available to researchers upon email request, see the ‘data availability’ statement at the end of the document. Importantly, all information that could identify participants has been removed to preserve anonymity. The analysis for this study including quotes and excerpts from participants is presented in the Findings and Discussion sections.

### Philosophical position

The present investigation was conceived and conducted through a pragmatistic lens [47, 48]. The primary reason for using pragmatism as the research paradigm, was to allow for an exploration of various stakeholder experiences to better understand the ‘practical information’ that can be used to inform future directions of the PMP (i.e., information from stakeholders on ‘what works’ and what does not work’ in the PMP). This ‘what works’ view is recognized in social and psychological research as being a worthy paradigm for researching organizational processes (in this case, an educational program) by focusing on the production of ‘actionable knowledge’ [47]. As well, the focus of pragmatistic research is to interrogate the value and meaning of the research (i.e., of what we are learning through this research investigation, and how can this be used practically in real life?) [47, 48], which is well-aligned with the overarching research focus of understanding the value and impact of the PMP for stakeholders, in order to investigate, understand, and improve the teaching and learning processes to enhance the quality of education.

The epistemological position of pragmatism is rooted in the idea that knowledge is constantly being debated or negotiated (i.e., individuals can experience action and change differently [47, 49]), which was particularly valuable for the present investigation. This was mainly because the authors placed value on exploring as many stakeholder perspectives as possible to try and identify the unique and diverse ways in which the PMP was understood (and perhaps, not understood) and experienced (and perhaps not experienced). Interpreting these experiences and perspectives through this lens allowed for ‘practical understandings’, which is seen as critical component of conducting a program evaluation [47, 50–52].

In aligning with pragmatism, the authors crafted the primary research question ‘*what is/are the perceived value(s) of a PMPs for SAs across various stakeholder positions*?’, and the research methods in a way that embraces and emphasises ‘*how*’ stakeholders of the PMP have learned about their perceptions of the program through their experiences. This focus on the ‘*how*’, meant that explorations of ‘*why that might be the case*’ was not at the focus of the research question [48]. As such, this focus on the ‘*how*’ further shaped the methodological approaches, and subsequently, the way the authors’ interpreted and wrote about the research findings (which will further be explained below.

### Theoretical underpinning

The present investigation used Utilization-Focused Evaluation (UFE; [52]) to guide and shape the research from its conceptualization to its interpretation. As with the priorities of pragmatism, UFE prioritizes evaluation for the ‘utility’ and ‘actual use’ of a process, program, tool, system, etc. and emphasises stakeholder experience(s) with its use [53]. In other words, it focuses on the evaluation of findings that can be used for reflection, decision making, and improvement. As noted by Patton [53], a UFE approach is appropriate for multiple purposes (i.e., formative, summative, developmental, etc.) and multiple research designs (i.e., naturalistic, experimental, etc.) focused on multiple aspects (i.e., processes, outcomes, impacts, cost-benefits, etc.) [53]. Put succinctly, “Evaluation processes include asking evaluative questions, applying evaluation logic, and gathering real-time data to inform ongoing decision making and adaptations” [53]. As well, UFE celebrates various stakeholder perspectives and seeks to elevate their voices in the evaluation process [52–54]. As such, the authors tried to include as many perspectives (e.g., students, staff, coaches, professors, teaching assistants) as possible.

Another key aspect of UFE, is that it is intended to promote a continuous learning environment where it encourages stakeholders (especially those within decision making positions) to reflect upon and learn from both the successes and failures of a program as interpreted by those who use the program. This creates a positive, feedforward cycle of implementing programming, evaluating programming, modifying programming, then re-evaluating, and so on, which is central to systematically studying and improving the teaching and learning process within the PMP.

### Methodology

A qualitative descriptive (QD) technique was used in this study as a strategy to interpret and report the data. In alignment with a pragmatic paradigm and UFE, this technique embraces the notion there are many different realities and those realities are changing and constantly being negotiated. QD has been recognized in multiple research disciplines as being an appropriate design to apply when embracing naturalistic perspectives (aka the research will aim to produce a relatively ‘straightforward’ description of a phenomena [55–59]. This was important to the researchers in the present study to stay as ‘close’ to the data as possible, while still producing an interpretive result that may help to inform the practice and delivery of the PMP [58, 60–62]. The authors recognize that a ‘straightforward’ description of the stakeholders’ experiences and perceptions is just one approach to understanding this research question, and that this strategy can provide a foundation for future investigations that include descriptions of the meaning or ‘essence’ of the experiences, which can further contribute to the broader theoretical and conceptual understanding of PMPs designed for supporting SAs.

### Participants

Participant were recruited using four separate approaches. For participants who were identified as ‘staff’ and ‘coaches’, the publicly available list of names, positions, and emails were accessed through the university website. All listed staff members and head coaches were contacted with their respective invitational email. For the varsity SAs (those in a mentor or mentee position), flyers were posted in common areas including the room where PMP sessions were held, the gymnasium, locker rooms, and hallways in the main building where the SAs practice and train. For the Professors, the PI scanned the list of core courses for first year students (those who would see the highest volume of first year students) and reached out via email to gauge interest in participation. For the TAs, a snowball technique was used to contact TAs of various first year courses via email.

Through these recruitment strategies, 44 people reached out to the primary investigator to inquire about study participation. Following an email correspondence with these interested participants, which included further information of the research project and the estimated time requirement, 30 of those individuals consented to participate in an interview, and all 30 interviews were included in the analysis.

The study included male-identifying (n = 16) and female-identifying (n = 14) participants from various stakeholder perspectives including first year SAs (also referred to as mentees; n = 9), peer mentors (n = 8), varsity coaches (n = 5), administrative staff (n = 5), professors (n = 2), and one teaching assistant (TA). Of the SAs (consisting of both mentees and mentors) in this sample, the participants spanned multiple male- and female-identifying sports including hockey, soccer, football, track and field, wrestling, volleyball, basketball, and rugby. All participants were from a singular Canadian institution, making this a convenience sample.

### Data collection

Semi-structured interviews were chosen to allow for a general structure while also creating the space for deeper exploration in participants’ responses (see S1 Table for a list of overarching and probing questions). To better understand the broader research question—*the perceived value(s) of a PMP for SAs across various stakeholder positions*, the first line of questioning (Focus A) was centered towards understanding the first year student experience from each of the stakeholder groups. Questions such as ‘*can you tell me about your first year student experience*?’, ‘*what are some of the challenges you faced*?’, ‘*what are some of the highlights of your first year*?’ were included. For those who were not SAs during their first year experience, questions such as ‘*what do you think are some of the challenges for SAs in first year specifically*?’ were explored to try and understand how those supporting first year SAs (including staff, coaches, professors, TAs) perceived the first year experience for student athletes in their respective roles.

**Fig 1:**
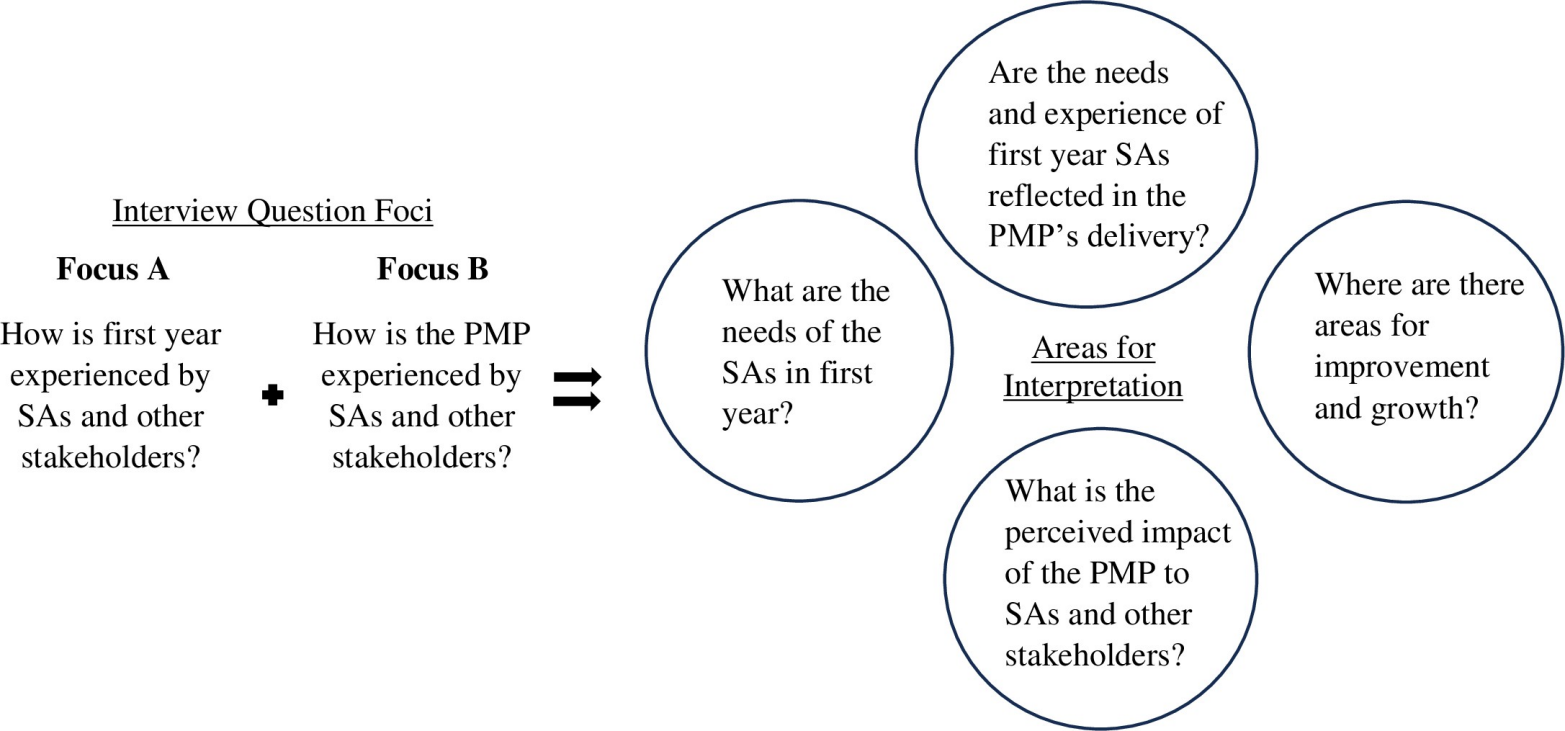
Visual depiction of the approach taken for the evaluation of the PMP using a Utilization-Focused Evaluation [52] approach.

Once that line of questioning was completed, the type of questions shifted towards the PMP itself (Focus B), to better understand perceptions of what the PMP was, the perceived goals of the PMP, the areas of strength, the areas of weakness, etc. for each of the stakeholder groups. In other words, the focus of the question moved from *how* first year was experienced, to *how* the PMP was experienced. Using this strategy allowed for focused discussions on whether the PMP accurately reflected the experiences of SAs in first year, and if not, what aspects required changes to better reflect the stakeholder experiences’. Please see Fig 1. for a visual depiction of the process and approach taken for the interview question and interpretation for the PMP evaluation.

### Data analysis

Interviews were recorded on two separate recording devices and transcribed verbatim and checked against the recordings. As noted above, participant codes were used to preserve anonymity. These codes are categorized by the stakeholder group they belong to, whereby PM indicates Mentors, ME indicates Mentees, P indicates Professors, T indicates Teaching Assistants, S indicates Staff, and C indicates Coaches. Of the 30 interviews, 29 of those interviews were in-person, with one interview conducted virtually due to COVID-19 restriction protocols. Interview length ranged from 10 minutes to one hour and 15 minutes (M = 25.33, SD±14.43).

We examined participant responses using a reflexive inductive thematic analysis (ITA) approach. To begin, the lead author refined and defined the primary research objective (*to better understand the perceived impact of the PMP through the lived experiences of the stakeholders*) and the aims (*to determine the PMPs value*, *its strengths and weaknesses*, *and its ability to accurately reflect the experiences and needs of the stakeholders*). The next step involved the lead author and secondary author immersing themselves in the data by listening to the recorded interviews, re-listening, reading the transcriptions, re-reading the transcriptions, and making notes along the way. Following this step, the first and second authors developed codes based on the primary and secondary authors’ interpretation of participant responses [63] using an ‘open-coding’ technique [64–66]. Upon completion of this process, the researchers independently examined random selections of interviews and performed a blind coding. The team of researchers then met to discuss the similarities and differences in their interpretations, which was followed by the creation and assignment of higher-order themes.

**Fig 2:**
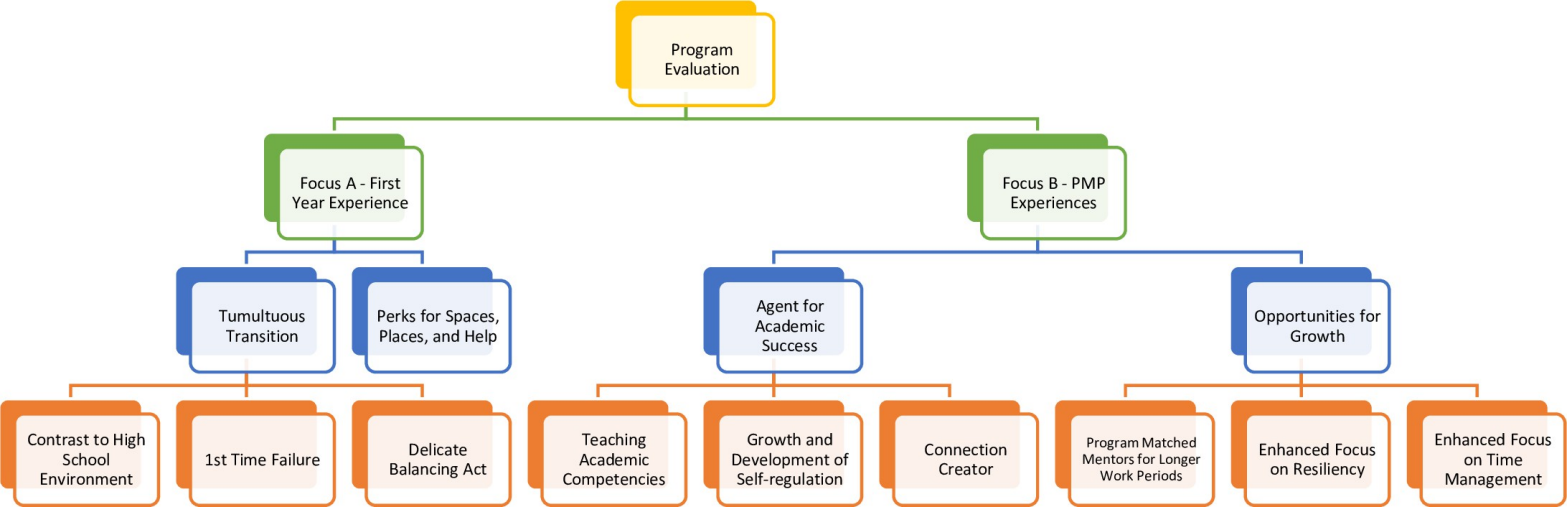
Visual depiction of the higher-order and middle-order themes separated by interview question foci.

As noted in the ‘Data collection’ section, the data were collected and analyzed, according to the interview question focus (A and B). In this sense, the thematic processes of applying higher-order and middle-order themes [64, 67] was separated by Focus A (‘*How is first year experienced by SAs and other stakeholders*’) and Focus B (‘*How is the PMP experienced by SAs and other stakeholders*?’). The authors then created a conceptual thematic map (Fig 2), which provides visualization of the interview question foci and the associated higher-order and middle-order themes.

## Findings

### Focus A) How is first year experienced by SAs and other stakeholders?

Through discussions with stakeholders regarding the first year experience, two higher order themes were identified. These higher-order themes included, a) tumultuous transitions, and b) perks for places, spaces, and help. These higher-order themes, along with their corresponding middle-order themes are presented below with supporting passages.

#### Tumultuous transitions

Participants of all stakeholder positions indicated a number of notable situations where their transition experience from high school to post-secondary schooling was anything but smooth. The nature of the specific situations and circumstances for those ‘bumpy’ times varied between the respondents, however, there were similarities in emotions and feelings elicited, and the contexts in which those feelings were elicited, which are categorized in three middle-ordered themes of a) contrast to the high school learning environment, b) first time failure, and c) delicate balancing act.

*Contrast to the high school learning environment*. Often, respondents could pinpoint time(s) during the first year experience when they recognized a notable shift in the learning environment. For example, the first time Mentor PM3 sat in a lecture hall with hundreds of students, was a memorable moment as he recalls “I wasn’t really expecting it to be like 500 people in a room, but then at the same time, I probably should have been because it’s University!”. This change in class size was noted in other responses as a surprising element of the new learning environment. When questioned further about how respondents believed a difference in classroom size impacted their learning, four students in mentor and mentee positions spoke about the lack of one-on-one support they had in the university environment compared to the high school environment. This sentiment was captured in a response by Mentee ME2:

I remember in high school, every teacher was always on your ass like all the time just like “oh yeah do your homework”, “you have to do this, you have to do this”, and they were always saying like “in university, no one’s gonna care about you, you’re just a number, you have to take care of yourself you have to be independent.”

When probed further on what ‘being a number meant’, Mentee ME2 shared that he believed the professors did not (and could not) have the capacity to provide individual support for students, as some lectures had over 500 students.

In addition to class size and the degree of independence, multiple interviewees reported feeling surprised by the effort needed to earn a ‘good’ grade at the university level compared to the high school level. Coach C5 recognized this shift in effort and expectations as he shared, “There’s a massive jump in terms of what’s expected of you and what an ‘A’ looks like at university compared to high school and I would guess, most people are coming out ill-prepared for what the academic rigors and expectations are at the university level”.

When asked to expand on what specifically changed from high school to university in terms of course work, Mentee ME9 recalled, “I just think tests in general are harder. I feel like high school was more like knowing stuff, but now it’s like knowing the little things and applying the little things to what you’re learning”. Mentee ME9’s reference to application of knowledge and ‘knowing the little things and applying the little things’ may indicate an important area of growth for the PMP. Specifically, it could be valuable to consider focusing the strategies taught during the academic sessions towards ways to ensure ‘learning’ has occurred while studying, such using knowledge check-ins (i.e., self-quizzing) or testing by teaching (trying to teach what is learned to someone or something) to help prepare for course assessments in a way that emphasizes this degree of detail.

When those in a teaching role (professor and TA) were asked to reflect on their experiences teaching first year students, and to provide their perspective on the difference(s) between high school and university level course work, each respondent referenced the standards, expectations, and nature of work deemed ‘acceptable’, at the university level compared to high school. Professor P1 believed there is a ‘shock factor’ when entering university, as many students do not possess the necessary learning habits that are required at higher levels of learning, which may contribute to a difficult academic experience. Professor P1 specifically stated, “They have just brutal, brutal, brutal study habits. And that’s okay!”. Professor P2 shared a similar sentiment when he recalled:

When they come to post-secondary, they’re not really equipped to make that jump, right? That understanding isn’t just being able to recite facts out of a book, right, or to recite things out of, you know, whatever source. It’s to be able to take a situation and apply what you know, to take a situation that you have never seen before and knowing what you know, make predictions about this situation, right? That’s ultimately what we’re asking students to do, and I find that a lot of a lot of students, they’re not equipped to learn in this fashion, they don’t quite know how to do that.

This statement from Professor P2 emphasizes what Mentee ME9 noted above, where there is recognition of the changes in the type of learning, knowledge and critical thinking required at the university level compared to high school. Importantly, Professor P1 further explained that he believes it is the responsibility of the institution and its membership to recognize the areas for improvement and to teach, development, and build the necessary competencies required. It was interpreted that he, alongside many of his colleagues, not only expects that students will enter with sub-par study habits, but that he even embraces it and works to build his course in such a way that supports students (such as offering additional office hours, providing practice tests in advance, providing ‘buddy-system’ support between students).

*First time failure*. Interestingly, many respondents discussed a critical point in their first year of studies when they experienced a ‘failure’ in a unique (and in some cases, traumatic) way. For many, achieving high standards was an expectation as many SAs excelled in both academic and athletic ways. It is perhaps the discrepancy in SAs’ ease of achievement or ability to achieve success (often evidenced by a poor assessment grade, or perhaps not making the ‘starting line up’ for competition) which was interpreted to be a significant, impactful, and memorable experience in the first-year experience. For instance, Mentor PM1 recalled:

I think my first midterm I got back was in math and I got like a 51, so something like that was really shocking to me and then, umm, a couple more midterms came back and were not too good, then you fall behind in the classes, and realize how much more work you have to put in at the university level to get success. That was a really tough transition. And then also athletically just realizing that you know, you’re no longer you know, ‘THE guy’ on campus.

Similarly, Coach C1 discussed her perspective of the SAs first-year experience and acknowledged that many SAs experience some type of adversity during this time “This is the first time in their lives, likely, that they’re not getting playing time and they’re not at home to get any support, and school is crazy and so it, it just becomes overwhelming…”. As Coach C1 notes, the compounded effects of living away from home, not earning the grades SAs desired, and not earning the playing/competition time they expected, can be very difficult for SAs to cope with. These feelings may be exacerbated if the student has not yet faced adversity of that nature before or had the opportunity to develop a toolbox of strategies to effectively cope during such situations.

When Staff S3 was asked about the challenges that first year SAs face (both from her experiences and from her vantage point as a staff member working with SAs), she responded:

Unrealistic expectations. It is their number one [biggest challenge]. I say that in terms of athletics, in terms of academics, in terms of supports, in terms of personal life skills, in terms of time management. I just, I think that unrealistic expectations is the resounding number one [biggest challenge]. Athletics are glorified on TV, right? If you watch certain movies like Glory Road or Coach Carter… everything’s really good, then it goes bad, and then it just it gets good again, right? And so umm the clip of climbing back up the hill in all of those sports movies is 2% of the overall film. In reality, that’s 98% of your experience at university.

When this topic was discussed with the participants in a teaching role, the notion of students being ‘protected’ from experiencing failure emerged, along with the potential consequences of such protection strategies. Specifically, TA T1’s quote captured this sentiment when she expressed:

…we just have a lot of student athletes who have potentially been sheltered from failure and have been probably taken out of bad situations, or you know, or have had a parent or guardian who has tried to protect them from having to really face up to, you know, not doing well in something or failing in something.

In Professor P1’s course, he discussed that this first time failure can be helpful for evoking a change of behaviour as he shared:

Sometimes it takes them failing the first test to realize…That’s okay! But they still get that grade, like they’ve still gotten that 40% on the opener, and they’ve thought, ‘even though it didn’t go right, it’s a wake up for my final grade’. Hopefully it’s a wake up call. For example, for the tests, I let them transfer fifteen percent of their grade from the lowest test of the highest test and the first test is worth fifteen percent so their first university test can be worth zero if it goes badly. And I like it because it doesn’t have to be the first test, because some students survived the first couple because it’s high school review, right and then they get rocked on test three or they get rocked on test three in semester two, you know? Whatever their natural abilities took them to a certain point and then the lack of study skills, the lack of university life experience, it’ll hit them at some point right.

Discussions such as these helped to illuminate that experiencing failure, or adversity, may be a common, traumatic, and perhaps, pivotal experience in the first year journey. For this reason, it will be important for the PMP to not only create a safety net for SAs when/if they do face personal failures/perceptions of failure, but to also create an environment that minimizes judgement on those failures, to better support the student through those times.

*Delicate balancing act*. The middle-order theme ‘*delicate balancing act*’ captures numerous comments from multiple stakeholder positions regarding the challenges of managing and balancing various social, emotional, psychological, and physical demands while at university. Specifically, stakeholders acknowledged that SAs must devote a large amount of time to both their academic and athletic activities, often combined with employment, volunteer work, caretaking, and transportation commitments. Not to mention, finding the time to fulfill personal, social, emotional, and spiritual needs. More specifically, when participants were asked what the biggest challenge has been in first year, nearly all respondents discussed ‘time management’. For instance, Mentee ME1 shared, “I think for me, that [time management] was my biggest struggle. Trying to find time to study because I have multiple tests during a week, to try and allocate that with also going to practice, and then for practice, sometimes I can’t go to because of class conflicts”. In this quote Mentee ME1 shares the challenges of having to manage the demands of her academics and athletics, and in some cases, to prioritize academics over athletic commitments when conflicts arose.

From a coach’s perspective, the workload (both academically and athletically) SAs have was frequently discussed. Discussions around the volume of work were often paired with conversations regarding time management skills as a means to manage and even thrive in such environments. Coach C4 stated,

There’s really no place to be a procrastinator as a student athlete and I think that’s beneficial for them [SAs] overall, but early on I think that’s a challenge because you’re so used to cramming last second, you’re so used to doing a paper the night before and you quickly realize in university that you cannot achieve good marks that way.

Mentor PM2 speaks of this as well, when he shared:

Time management and organization. It’s the biggest thing. Once you can organize a proper schedule, it’s so much easier, but when you come in, everything hits you at the same time. If you have four or five courses, you have four or five different syllabi, then you got your practice schedule, and you got to put it all together, and then there’s the clinic, and then there’s [the PMP] you have to schedule in for. So there’s a ton of different things you have to do and that is really the hardest part just putting it all together before you can get into it because if you start off on the wrong foot, it’s really hard to catch up later and organize yourself.

Mentee ME7 shared a similar sentiment, but also discussed her perception of the advantages SAs in their time management skills in her statement,

I have friends who are not athletes and because they have like so much time in the week, they’re like putting off doing stuff even later because they have a lot of time. Where it’s like we [SAs] have to stick to these schedules and like when you do have an hour like you do stuff, you do schoolwork in that hour.

In her quote, Mentee ME7 frames the busy schedule that SAs have, as a potential advantage for achieving academic success. When further probed on the topic, she explained that SAs may exhibit superior time management skills compared to many non-SAs because their environment is so scheduled (including classes, practices, competition travel, etc.), further echoing the sentiments shared by Mentor PM2 and Mentee ME1.

#### Perks for spaces, places, and help

The second higher-order theme relates to participants’ responses regarding the value of being a SA during the first year experience. Participants from multiple stakeholder positions commented on the ‘perks’ of being a SA in such a way that afforded them spaces on campus, such as a designated area to practice, attend strength and conditioning sessions, obtain athletic therapy, and attend PMP sessions. For example, Mentor PM7 explained the varsity SA community gave her a sense of belonging and created a space and a place for like-minded individuals to form relationships as she shared:

It was really challenging for me in my first year to make friends right off the bat. It was something I forced myself to, but I was terrified every single minute because my anxiety is always so bad. But as a varsity athlete, you come into this family and immediately you’ve got people who are supporting you and to go and like hang out or study together and you never really question where you fit in, and I think on a big campus like that or like this, rather, it’s important that you have this place where you kind of just immediately fall into.

Coach C1 shared similar sentiments to PM7 in acknowledging the comradery developed within the SA group in the following quotation:

I also think that the connections even just in terms of the fun environment where all student-athletes from different sports are meeting and or you know working in the same space umm that you know, also increases connections among the [institution’s] community.

It was interpreted from quotes such as Coach C1 and Mentor PM7 ‘s that the varsity SA environment, promoted connection building both between-team(s) (e.g., soccer teams and volleyball teams in the same space) and between-year (first year SAs and upper-year SAs together), perhaps highlighting the perceived importance of having PMP sessions with multiple sports in one session, instead of the more traditional ‘team study hall’ approaches.

The social connections built, and opportunities for those social connections to be had, was viewed by one participant as one of the main privileges of being a SA, especially during first year, as shared by Mentor PM6 in his response:

I think that connection part makes a big difference like between someone who’s not doing well and has no connections and someone who’s not doing well and has connections, usually that person who’s not doing well and has connections is gonna stay around. It’s that person who has no connections and no [PMP] and isn’t doing well, they’re not gonna be here.

In a similar sense, Mentor PM4 explained:

For me, the purpose of [the PMP] would be to make first year students become more successful than them being on their own. You know, because not, not every first-year student has that, you know, someone to go to, it’s like a shoulder to lean on during your first year.

These comments were interpreted to mean that being part of the SA community, and having access to supports like the PMPs can be a powerful vehicle for relationship-building which can further support students when challenges arise.

### Focus B) How is the PMP experienced by SAs and other stakeholders?

This section captures the higher-order and middle-order themes for the line of questioning that was focused on understanding stakeholder experiences in the PMP, including a) that the PMP was understood to be an agent for academic success, and b) that there were multiple opportunities for areas of growth.

#### Agent for academic success

This higher-order theme represents focused commentary provided by respondents from all stakeholder positions regarding the academic content delivered through the PMP and the perceived value it had on academic success. Three middle-order themes were identified, capturing the specific topics and aspects of the PMP believed to influence academic success including: a) the teaching of academic competencies, b) growth and development of self-regulation skills, and c) creating connections to people and places on campus.

*Teaching of academic competencies*. A portion of the PMP’s delivery is focused on teaching fundamental learning skills. These skills were delivered in a curriculum format and were varied in terms of their focus and period of delivery (i.e., an instruction session on adding and dropping courses is presented early in the semester before skills for effective note taking are taught). Multiple participants referenced the learning of such skills during the PMP sessions and acknowledged its value and applicableness both within the university environment, and beyond. For example, Coach C2 discussed his appreciation for the PMP offering these learning skill seminars/workshops for the athletes he coached in the following excerpt:

To be able to learn different skills like ‘how to read before lectures’, ‘how to take notes’, ‘how to study for and take multiple choice tests’, all of those kinds of skills are critically important that allow them to be successful in whatever path they choose….

In this quote, Coach C2 outlined some of the specific session foci offered in the PMP, drawing special attention to two separate teaching areas–preparing for a lecture (pre-reading of materials) and strategies for reducing test-taking anxiety. He also shared his perception of the value of those skills being taught in various domains. Specifically, he noted the value in catering these skills to meet program-specific demands (e.g., how to study for a biology course exam, may look different than a social studies exam), which may shed light on the notion that this type of focused skill development is a valued aspect of the PMP’s curriculum.

Along with Coach C2, there were others (SAs and staff) who discussed the different skill building workshops presented in the PMP sessions. Mentee ME3 commented, “You guys [the PMP] cover a lot of good stuff, like how to study, how to take better study breaks, and also like the ‘how to interview’ stuff. It will be good for like when I go home because I need to make money at some point…”. In this example, Mentee ME3 referenced two topics of the PMP curriculum delivery, including study strategies and career preparation skills (such as interview practice and cover letter/résumé creation) that she believed would be valuable.

*Growth and development of self-regulation*. This middle-order theme represents stakeholder’s recognition of how the PMP provided opportunities to learn about the value of, and to practice, self-regulation in the context of SAs’ learning. In this sense, self-regulation refers to the self generated thoughts, feelings and actions that are planned to the attain a personal goal [68]. Stakeholders from multiple roles expressed the importance of learning about self-regulatory behaviours such as metacognition (e.g., the act of thinking about one’s thinking), motivation to meet standards (e.g., strategically determining a goal, devising a plan, and taking action accordingly), and self control (e.g., the ability to prioritize certain things as certain times). Coach C2 draws particular attention to the value of self-regulation in his own coaching and the importance of this practice in the PMP in the following quotation:

I think the awesome conversation that [the PMP] facilitates with a lot of kids is like okay, you got this mark, doesn’t matter if it’s good or bad, how did we get here? Like what did we do? Did we do it with quality? What would we do differently? What lesson can you take? Did someone else prepare the same way and get a different mark, and why? … They will eventually look back and be like, I’m really glad my coach and [the PMP] taught me how to learn, how to be able to dive deep in that way, how to self-assess, and how to be self-reflective.

In this quote, Coach C2 shared the perceived value of the PMP in its ability to facilitate an environment that promotes metacognitive practices. Coach C2 also explained that he tried to embed such practices into his coaching when discussing academics with the SAs. This is a powerful sentiment which may shed light on the degree of alignment between departmental units (in this case, sport coaching and academic coaching) allowing messages such as the importance self-regulation in the context of learning, to be echoed from multiple leadership roles. This also speaks to the many ‘hats’ coaches wear at the collegiate level to support SAs from various perspectives (e.g., academically and athletically [among other aspects]).

At the time of the study, there were particular sessions of the PMP dedicated to teaching self-regulation as a concept and skill, and there was time allocated to developing and building upon students’ self-regulation practices as the year progressed. For example, during a student’s PMP session, journalling, logging, and sharing/story-telling from peer-to-peer and peer-to-mentor was encouraged. It was during these reflective practices, that students were to consider their own approach(es) to learning, and learning process(es), and to self-evaluate behaviours that were helpful (i.e., strategies for staying focused) and hurtful (i.e., catastrophizing a poor grade) in the students’ learning journey. Professor P1 noted the value of such self-regulation practices (including the specific skills of goal setting and self-reflection) and how he has chosen to integrate those practices in his own course delivery. Professor P1 explained his perspective on how students can benefit from integrating specific, measurable, and attainable goals in the learning process:

Reflecting after a test, after an assessment, and having them set a goal for the next one… even just getting them to go to there and go to test-pickup, or whatever, right, is a huge step…

These findings illuminate that self-regulation skills are taught and practiced in other areas of SAs’ university experiences beyond the sport and PMP highlighting the value of such skills at the post-secondary, and collegiate sport levels.

*Connection creator*. In addition to the academic competencies that were believed to be developed in the PMP, the idea that the PMP fosters connections to people (e.g., between mentors and mentees, and between staff and mentees) and to places (e.g., resources on campus), was another frequently discussed aspect of the PMP during the mentor and mentee interviews. For example, Mentee ME1 noted: “I’ve developed such a great relationship with my mentor and I feel like it’s kind of cliché, like I feel like we’re friends. Yeah, it’s so easy to talk to her, but not even for just school stuff, just like personal stuff”. Similarly, Mentee ME8 shared “my [PMP] group, is more like social. Like we do work, but it’s more of like a social thing too so we always talk about like stories and stuff what happened to us. I see it more as like the study-social kind of program”.

Mentor PM7 noted the importance of building an environment and culture that supports social connections, where students feel comfortable asking for help when/if they need it. For instance, Mentor PM7 shared,

It [the PMP] really makes a different kind of family setting where students can ask for help knowing their mentors will probably have the answers, if they don’t, then the [PMP] Coordinator knows the answers, and it’ll just kind of keep going up and up because of their connections.

This sentiment was shared by Staff S2, who stated:

And as we transition them into university, and some of our first years come even before classes begin for training camps and all kinds of things, it’s critical that they get grounded before classes start. There’s notion of creating a sense of community and ensuring that they have a foundation of transitional skills. Without those two things, I personally believe there’s greater opportunity for them to languish and really struggle at finding their footing. So through the peer through the peer mentoring piece, I mean, they have a community their engaged in

In addition to the mentee and mentor connections, multiple participants discussed the importance of the PMP in building connections to resources. Multiple stakeholders referenced specific workshops they benefitted from, which were led by university experts (e.g., academic advisors, librarian, career services). For instance, Mentor PM7 explained, “I think [the PMP] touches on a really wide variety of topics, be it learning skills, or where to go for different resources. The library presentations I think are super important because students are generally afraid to go into the library and start like actually looking through books”. In this example, Mentor PM7 draws specific attention to the value in having librarians attend sessions to discuss their diverse and extensive offerings such as strategies for locating resources, which may be daunting to first year students. In recognizing the value of the PMP in being a ‘connection creator’, it will be important to continue to build connections on campus in an effort to spread awareness and reduce as many barriers as possible for students when accessing such resources.

## Opportunities for growth

As a key pillar of UFE, interview questions were directed towards identifying gaps and areas for growth and improvement within the PMP such as ‘*if you were in charge of the PMP*, *what are some things you would change*?’. These questions were asked to all stakeholders to invite discussions on the ways in which the PMP could be modified to better meet the needs of the SAs during their transition into post-secondary, and throughout their first year. Through the authors’ analyses, the following middle-ordered themes were identified, a) the need for program-matched mentors and optional, longer work sessions, b) offering enhanced resilience strategies, and c) allocating a greater focus on time-management strategies and building good habits.

### Program-matched mentors and optional, longer work periods

Multiple students (in both mentor and mentor roles) acknowledged the benefit of the dedicated time that the PMP offered SA to work quietly on their schoolwork. This work time was perceived to be even more valuable for those mentees who had program-matched mentors (i.e., a mentee studying math would be matched with a mentor studying math) as mentees were able to ask their mentors subject-specific questions. For instance, Mentor PM8 provided a personal story of how valuable it was for her to have a program-matched mentor when she was a mentee. She explained, “I switched my program my first year. So that was way easier having that support like my mentor was in kinesiology and my mentor in training, so that helped me as well, like it was the transition, I didn’t really know what I wanted to do, so I had an idea but she [mentor] helped me a lot, like they [mentor and mentor in training] really helped me”.

For two mentees who did not have a program-matched mentor and mentor in training, they raised the point that their mentor was unable to answer certain course-related questions because their mentors were not familiar with that subject matter. This appeared to be particularly evident for science students who had mentors who were in the arts and humanities. Mentee ME6 shared his surprise when he became a mentee, and that his mentor was not in the same program of study. He explained:

I thought it [the PMP] was gonna be more tutoring kind of thing yeah. I mean, also at the same time this is little harder for me because I’m in engineering, so I have all the sciences and stuff, and a lot of the mentors here are not necessarily in that region…So like when there’s not very many tutors, it’s kind of the same in like study hall too, cause I don’t have [engineering] tutors in study hall…Don’t get me wrong, my mentors are like really good at like helping me set up schedules, what to study, when to study, how to prepare for it and stuff

Importantly, when asked ways to improve the PMP, Mentee ME5 explained that if he was a mentor, he would want to be in the same program so that he could better support the SAs he mentors, which raised discussions around ways to not only support the mentees, but also the mentors in their mentorship experience. He shared specifically that, “I’m in like mechanical engineering, right? If I have a mentee who’s in mechanical engineering or just any engineering in first year, because first is very general, and I’d be way more helpful to them”.

At the time of the interviews, implementing program-matched mentors was relatively new to the program, so it was likely that mentors who would have been through the PMP as a mentee in their first year, would not have had a program-matched mentor. It was only more recently that this was implemented when matching mentors, and as noted in the introduction section, the first priority for matching mentors and mentees at the beginning of the school year, is by schedule availability, with a *secondary* focus on matching by academic program. Of course, there are potential scheduling barriers for prioritizing course-matched mentors, which may mean reconsideration of the six times offered for the PMP to take place. It would be worth considering what the cost-benefit would be for offering more sessions offered within the week, with fewer mentors and mentees in the groups. If the space and supervision allows, or perhaps finding ways to offer separate, additional, and perhaps optional sessions that are work-focused with course-matched mentors, it may help to address this area for improvement.

Relatedly, three mentees asked for an option to have a longer work period during the PMP sessions. For example, Mentee ME8 noted: “…it [the PMP sessions] could be a bit longer because after like the first little bit of the presentation when we actually do work it’s only about like an hour maybe less left to do work in the work session and sometimes it’s hard to get enough like productivity, like productive work done”. In a similar vein, Mentor PM6 expressed, “sky’s the limit? I’d make it [the PMP] two hours to be honest. Then you could have like a full hour check-in and then a full hour study. I think that would be helpful.”

While this was interesting to hear, some notable space and resource constraints may come into play. It could be worth exploring options for other peer-to-peer groups to arrange quiet study time. This could be done by team, or even by program as many programs have dedicated work spaces for booking, as the rooms available for the PMP are also used by others including staff, coaches, community rentals etc., making it difficult to increase the booking time for the PMP.

### Resilience-focused discussions and strategies

Interestingly, only those in non-student positions (including those in staff, coaching, professor roles), discussed the need for resilience awareness and training within the PMP as a future area for growth and improvement. Staff S4 stated “if we don’t hook them in the first year around the transition and create that emotional attachment and start to develop some resilience and self-accountability, then the rest of what we’re trying to do goes out the window!”. Staff S3 also highlighted resiliency, when asked the probing question ‘what do SAs need more of to succeed in first year?’ by sharing, “I think that a lot of our young people are ill-prepared when it comes to what it means because to succeed…they’re not equipped, they need resiliency, and to try and be as proactive as possible”.

Similarly, when discussing ways to improve the PMP, Coach C1 shared:

…you know, now it’s getting into like mid to late October, now things are, you know, maybe a bit more serious and now they’re not getting playing time right and so again, this is the first time in their lives likely that they’re not getting playing time and they’re not at home to get any support and school is crazy yeah right and so it just almost becomes overwhelming and depending on their experience with resiliency or perseverance, they almost don’t even know where to start. And that’s where I think, you know, having the having access to support but also, just being more informed on the supports that are available to them… I still think that based on the student athlete that we’re getting right now, I think that if we wanted to add in another layer, I think, whether it’s having maybe more resources, or support on how to build resiliency, that would be valuable.

These examples raise important questions around how resilience skills might shape the transition period to university and how it might influence their overall first year experience. It also raises questions around what resilience means for various stakeholders in the context of academic success and how resiliency can be assessment, development, and practiced within the context of a PMP–all questions deserving research explorations in future work.

### Greater focus on time-management strategies and good habit forming

As noted above, nearly all stakeholders illuded to the fact that time-management is one of the most challenging aspects, and most important tools for navigating first year. When asked what areas the PMP could grow in, a common response from all stakeholder positions was to enhance and augment the delivery of time management teachings. While this is already embedded in the PMP as a topic for teaching in the curriculum in the first few weeks of school, it would appear students could benefit from having a greater focus on this topic at multiple points throughout the year. For instance, Mentor PM4 stated “I’ve gotten really used to being able to be good at time management, but I think [the PMP] should really focus on implementing that and going over it a lot more”.

When probed further about ways to augment teachings around time management, Professor P1 suggested that the PMP should focus on creating discussions around, and providing information on aspects of time management, as he shared:

“How do you manage your time? How do you set a goal that’s actually going to work? What won’t work? How do you get out of that habit? And not just write a test and get results back and think, ‘Oh no, I failed. Next!’ And so we need to try to get them out of that, breaking out of those bad study habits.”

Coach C3 shared a similar perspective when she expressed, “…it comes down to time management and how to section off your time how to be diligent within that time. And that’s something that I don’t know if you can teach in a classroom. Maybe it’s a bit of creature of habit there, but something that continues to challenge this generation, as there’s more and more distractions that keep creeping in, I think it’s important to try and train good habits”.

It will be important for those in decision-making positions to consider ways to teach, train, and develop habits that support effective time management for SAs. A future area of exploration could be to explore ‘best/better’ approaches and practices, which might include bringing upper-year students in as speakers and inviting professors and TA’s of first year courses to give personalized and experienced insight into some of the ways students can mitigate challenges.

## Discussion

This program evaluation was designed to determine the value(s) of the PMP in supporting students in their first year experience and also to determine whether the goals and intentions of the present PMP under investigation met the needs of its stakeholders. In the following discussion, we examine the findings and their significance in the context of the PMP, and consider the feedback from stakeholders regarding the ways in which the PMP can be improved upon.

### Implications of findings for the PMP

The data collected from 30 semi-structured interviews provides insight into the effectiveness of the PMP and offers valuable information on the specific aspects that are aligned with the intended goals for SAs. Findings indicate that the PMP provided opportunities to learn academic competencies, develop and practice self-regulation skills, and build important connections to both people and places on campus. For these reasons, the authors posit that the PMP met most of its foundational goals. As referenced earlier, the PMP’s handbook states the goals of the program are to: a) provide a consistent environment for first year SAs to learn and develop academic competencies through an established curriculum; b) create a safe and comfortable space that fosters deep social connections; c) to connect students to on-and off-campus resources, and d) to have fun. Based on the present findings, at least three of these goals were identified from discussions with stakeholders; specifically, the teaching of academic competencies and meaningful connections to people and resources.

Our data also indicates that the first-year experience is a particularly tumultuous time for SAs. This was illuminated through stakeholder discussions around specific (and common) areas of challenge. These challenges included recognizing (and being surprised by) a change in the standards required for earning ‘good’ grades, facing a struggle or even failure for the first time, and trying to balancing and manage the workload required to remain a student and a varsity athlete. Using these important findings as a guide, we triangulated the information gleaned from stakeholder interviews, with the PMP’s curriculum and its proposed goals and intentions, and concluded the PMP reflects and supports the lived experiences of first year SAs. Specifically, stakeholders believe that first year academic standards are notably different compared to those in high school and that there is a dramatic shift in both the learning process and the expectation of instructors when assessing students’ performance. In this case, teaching academic competencies through the PMP’s curriculum will continue to benefit SAs in a way that helps them develop the fundamental skills needed to succeed academically in first year. This approach has been recognized by others in the field (for more information see [69–72]) as being an important component of helping students thrive in the transition period between high school and post-secondary studies, with a particular emphasis placed on teaching fundamental learning skills for students that are developmentally appropriate while also building competence in those areas.

It was also interpreted that integrating learning skills workshops that extended beyond the classroom experience (such as job interview preparation workshops and financial literacy) is well-received by the first year SAs. Furthermore, teaching fundamental skills (e.g., effective note taking and studying strategies) and self-regulation skills were understood as important and helpful components of the PMP, and should be continued in the program’s curriculum and delivery to help foster success. For these reasons, the integration of such teachings will continue to be delivered in the PMP’s curriculum and will remain a central focus for continued research and development to further enhance the quality of such teachings.

The data also demonstrates that the opportunity for SAs to connect with peers both within and beyond their sport and academic year was perceived to be a significant aspect leading to the effectiveness of the PMP. This finding aligns with others in the field who recognized meaningful relationships are believed to foster and grow an individual’s capacity for academic success as students who interact frequently with their peers in educationally-purposeful ways are more likely to engage with their learning in deeper ways [60–62, 73]. As such, creating both formal and informal opportunities for students to interact and develop meaningful relationships will continue to be a focus for the delivery of the PMP.

Moreover, these meaningful relationships may become even more critical for students experiencing difficult transitional periods such as the one between high school and university [61, 62]. As recognized for the participants in the current sample, first year came with situations of adversity. With many students reporting experiences relating to ‘failure’, along with challenges socially and emotionally, it appears critical to provide structured and formal social and instructional support (and perhaps even in an augmented way) in the PMP. As noted by Pascarella and colleagues [74], students with meaningful social connections are more likely to seek help when needed. Moreover, Krause and Coates [60] illuminated that students who have a stronger social network often have increased feelings of engagement with the institution which may lead to increased retention and graduation rates and, enhanced student experiences [60]. With this in mind, and in alignment with the present findings, the PMP can (and appears to) be an important and meaningful space and place for connection building, and thus will continue to be an integral component of the PMP programming.

As mentioned, a critical component of this investigation was to discern the strengths and weaknesses of the PMP. It was identified that a key area of growth to enhance the PMP included augmented sessions focused on time-management strategies and resiliency. As nearly all stakeholders in the present study discussed the critical nature of being able to manage one’s times as a SA, it draws attention to the value it has as being a central focus of the PMP’s curriculum. This finding aligns with work done by Marizu [75] where SAs at the University of Illinois were asked about advice to give to other incoming SAs, the most common response was regarding effective time management [75]. While this topic is already a key focus at the beginning of the school year in the present PMP’s curriculum, it would appear students could benefit from having a greater focus on this topic, and perhaps at multiple points throughout the year. It is important to note, however, that Krause and Coates [60] draw attention to the subjective notion of ‘balance’ and how much more work is needed to understand the nuanced and complex relationship with student development and student success. The authors recognize that on the one hand, having limited, non-structured time can present challenges to academic success, but on the other hand, it can encourage students to be efficient with their time and adopt the ‘work smart not hard’ type of mindset and practices. As seen by MacNamara and Collins’ [61], the authors recognized SA’s time management skills along with their ingenuity to regulate the impact of performance-related pressure, might have enabled them to effectively cope with the competing demands during the transition to university. This could be a particularly interesting avenue to explore with the SA demographic at the present PMP’s institution, as a contextualized approach to teaching and practicing ‘time management strategies’ may be more relevant and impactful.

As well, with a growing interest in the connection between resilience and success across multiple domains like education, sport performance, industry, and the workplace, etc., it was perhaps not surprising that this was identified as an important area for the PMP to continue to deliver, but also to augment in its future iterations. This suggestion is timely, as work in the field of education has illuminated the value of teaching resiliency and resiliency strategies, and the connection between overcoming setbacks and challenges, to help them bounce back from experiencing failures, adapt to new learning environments, persevere through difficult courses, and to manage stress and anxiety effectively [76, 77]. This may be especially true in light of the COVID-19 pandemic [78–80]. As resilient individuals are better at seeking support, maintaining a positive outlook, and managing their emotional well-being, this may not only help them with their academics, but also life beyond the academic institution and towards career readiness to prepare students for the challenges they may face in their future careers and help them build the skills needed to thrive in the workforce.

Finally, stakeholders raised important suggestions for improvement for increasing the number of program-matched mentors to help mentees during the quiet work. With multiple mentees and mentors asking for more and longer (and perhaps optional) work periods, we believe that there is value in including a work period for SAs during PMP sessions to complement the curriculum delivery component and the social interaction component of the PMP.

### Limitations and future directions

From a methodological perspective, the authors acknowledge that the use of UFE may make the investigation particularly vulnerable to biases as it emphasizes utility and stakeholder preferences, which can sometimes reflect the beliefs and desires of those who are already in places and spaces of power and authority, at the expense of those who may be under-represented in certain spaces. On a similar note, the use of UFE has been recognized for downplaying the broader socio-political and contextual factors at play. We recognize this, and acknowledge that this might have tension with more traditional evaluation and qualitative approaches and methodologies. Future work would benefit from approaching the research questions (and other related questions) in ways that delve deeper into the ‘*why*’ from multiple dimensions (i.e., sociological, cultural, political), to better understand complex underpinnings student success. To avoid this, the authors tried to be as careful, thoughtful, and intentional as possible in creating and implementing the study design while trying to balance the needs of the stakeholders (i.e., students and non-students) and those using the findings from the evaluations (i.e., the decision-makers).

Additionally, the nature of conducting interviews creates the potential for the impact of the social desirability bias to influence participant responses. In this sense, the interviewees may be more likely to respond in a way they believe the interviewer would prefer. Connectedly, the fact that the interviews were all conducted in the final months of the school year, may have led to a recency bias, whereby respondents are more likely to recall memories that are more recent. To decrease these biases/influences the primary researcher encouraged the respondents to be as open and honest as possible, and to think back to earlier time points in the school year that might not have come as readily to mind.

From a sampling perspective, compared to the number of students, staff, and coaches, those belonging to the Professor group and the TA group were largely underrepresented. While the PI attempted to gather as many perspectives as possible from each of the identified stakeholder positions, we recognize having only three participants from these sub-groups means that many voices were not heard. In a similar sense, the authors want to acknowledge that demographic information such as race, ethnicity, sexual orientation, disability, etc. was not collected nor discussed in the context of this work. This was a missed opportunity to explore the ways in which the PMP acted as a facilitator of, and a barrier to, students from underrepresented groups. Future work could benefit from including this lens to the research investigations as they are inextricably linked and could ultimately lead to the enhanced support for all students and their unique identities.

Acknowledging the current investigation was conducted at a singular institution means the project can be understood as a case study and cannot be interpreted widely across time and space and therefore, may not be not generalizable. This may be even more pronounced as the nature of the way students learn, experience first year and, therefore, the PMP, has changed in light of the world-wide pandemic form the COVID-19 virus. For example, with safety protocols in place in educational institutions in Canada, most programming was moved to an online format, and offered in a hybrid design following the return-to-campus. This may make applying the findings to the virtual experience difficult and potentially not relevant in some spheres.

The authors appreciate and respect that conducting an effective program evaluation requires multiple approaches (both quantitatively and qualitatively) from multiple guiding theoretical paradigms, with various methods and approaches. The choices made for the present program evaluations’ methodologies and methods are, admittedly, just one avenue for learning how to better support students, and will ideally act as a launch pad for future research. Moving forward, researchers could benefit from applying qualitative, quantitative, and mix-methods approaches to explore questions such as ‘*in what ways might PMPs help SAs*?’, ‘*what are the costs and benefits of implementing PMPs in an institutional setting*?’. Furthermore, researchers could seek to extend investigations with students who are not SAs. This would facilitate a more diverse perspective on the student athlete experience and allow for opportunities to develop specific resources for student supports.

## Conclusion

Our data demonstrate that the lived experiences of SAs at the present institution, during the transition from high school to university offers a range of experiences, perceived to be both positive and negative. Through discussions with stakeholders, it was perceived that PMP is an important agent for academic success, a place for social connection to take place, and an opportunity for students to access resources both on-and off-campus. These findings satisfy three out of the four goals of the PMP, and for that reason combined with rich commentary through the interview process, the authors believe the program is designed in an intentional and meaningful way that positively impacts the SAs in first year.

Specifically, the PMP was understood by the stakeholders to be a vehicle for teaching academic competencies, to help foster relationships and to build connections and to provide students with the resources they need for a successful transition to university. The respondents addressed the key areas of concern in the first-year experience including academic difficulties (e.g., contrast to high school learning, first time failure and difficult balancing act), and social struggles. Areas of improvement emerged from these findings which include enhancing the content and applicability of time-management strategies, offering program-matched mentors, and increasing teachings on resiliency. It is the hope that by applying these findings to the present PMP that the program will further support SA needs, and provide a more positive, accessible, inclusive, and meaningful first year experience.

## Supporting information

S1 Table List of main questions and their associated probing questions. (DOCX)
