# Supplementary material for: “A shoulder to lean on during your first year”—An exploration into a Canadian post-secondary institution’s peer mentor program for varsity student athletes
Source: PLoS One. 2024 May 8;19(5):e0298806. doi: 10.1371/journal.pone.0298806 (PMC11078383; doi:10.1371/journal.pone.0298806)
Supplement: S1 Table — (DOCX) [file pone.0298806.s001.docx]

# **S1 Supporting information 1. List of main questions and their associated probing questions**

#

| Main Question | Probing question | |
| --- | --- | --- |
| Tell me about your first year student experience | - What are some of the challenges you faced as a SA? - When did you first notice that was a challenge for you? - How long did that remain a challenge for you? - What are some of the things that could have helped you during that challenging time? - What are some of the highlights of first year - What made them such a highlight for you? - What/who supported you to achieve/experience that highlight?   **If not a SA**:   - What do you remember about your first-year experience? - What are some of the challenges you faced as a student? - When did you first notice that was a challenge for you? - How long did that remain a challenge for you? - What are some of the things that could have helped you during that challenging time? - What are some of the highlights of first year - What made them such a highlight for you? - What/who supported you to achieve/experience that highlight? - Thinking about the first year SA experience, what do you think are some of the challenges for SAs in first year specifically? | |
| What is your relationship to the PMP? | - What role did/do you have? - Are you a current or previous student athlete? If so, what team were you on? - How long did you spend connected to the PMP? | |
|  | **If no direct relationship**:   - How did you hear about the PMP? - When did you first hear about it? | |
| What does the PMP mean to you? | - What are the intentions of the PMP? - What are the goals of the PMP? | |
| What areas can be improved within the PMP? | - What would you like to see changed? - What would you like to see augmented? - What would you like to see reduced? - If you were set in charge of the PMP tomorrow, what is something you would like to do to support students? | |
| Is there anything else you would like to share about the PMP or the first-year experience? | |  |
